# The Impact of Methylphenidate on Sexual Functions: A Systematic Review of Benefits and Risks

**DOI:** 10.3390/ph18050718

**Published:** 2025-05-14

**Authors:** Rafał Bieś, Zuzanna Szewczyk, Anna Warchala, Ewa Martyniak, Marek Krzystanek

**Affiliations:** 1 Doctoral School, Department and Clinic of Psychiatric Rehabilitation, Faculty of Medical Sciences, Medical University of Silesia, Ziołowa 45/47, 40-635 Katowice, Poland; 2 Medical Students’ Association, Department and Clinic of Psychiatric Rehabilitation, Faculty of Medical Sciences, Medical University of Silesia, 40-055 Katowice, Poland; s86246@365.sum.edu.pl; 3 Department and Clinic of Psychiatric Rehabilitation, Faculty of Medical Sciences, Medical University of Silesia, Ziołowa 45/47, 40-635 Katowice, Poland; awarchala@sum.edu.pl (A.W.); emartyniak@sum.edu.pl (E.M.); m.krzystanek@sum.edu.pl (M.K.)

**Keywords:** methylphenidate, libido, sexual function, sexual disturbances, sexual arousal, ejaculation, quality of life, efficacy, tolerance

## Abstract

**Background:** Methylphenidate is a psychostimulant that enhances dopamine and norepinephrine neurotransmission through the mechanism of reuptake inhibition at the synaptic cleft. Studies indicate that sexual dysfunction is prevalent among psychiatric patients. The objective of our study was to assess the impact of methylphenidate on patients’ sexual health, identify specific types of sexual dysfunction, and analyse the correlations between psychiatric disorders, treatment dosages and durations, and the presence of sexual dysfunction. Additionally, we aimed to evaluate the prevalence of improved sexual function resulting from methylphenidate use. **Methods:** A systematic literature review was performed using the PubMed database in accordance with PRISMA guidelines. The initial search yielded 186 articles, of which 14 met the inclusion criteria and were analyzed. Clinical studies involving changes in libido, erectile function, ejaculation, semen quality, and sexual behavior due to methylphenidate were reviewed. **Results:** The findings indicate that methylphenidate can have both negative and positive effects on sexual function. In some patients, particularly those with psychiatric comorbidities, methylphenidate was associated with decreased libido and ejaculation disorders. In other cases, especially in individuals with preexisting dysfunctions or on low doses, it appeared to enhance sexual arousal and performance. **Conclusions:** Methylphenidate may influence sexual function in complex ways depending on individual patient profiles and treatment variables. Clinicians should be aware of these potential outcomes and consider sexual health as part of the therapeutic discussion when prescribing methylphenidate.

## 1. Introduction

Methylphenidate is a psychostimulant that enhances dopamine and norepinephrine neurotransmission through the mechanism of reuptake inhibition at the synaptic cleft. At sufficiently high doses, it may also modulate μ-opioid receptor activity [1]. It has been used clinically for over 60 years [2]. This drug exists as two enantiomers, d-threo-methylphenidate and l-threo-methylphenidate, with the former having been developed as a standalone medication for the treatment of attention-deficit hyperactivity disorder (ADHD) [3].

By blocking the reuptake of dopamine and norepinephrine into the presynaptic neuron through the inhibition of the dopamine transporter (DAT, SLC6A3) and the norepinephrine transporter (NAT, SLC6A2), methylphenidate increases extracellular synaptic concentrations of these neurotransmitters. These, in turn, may bind to their respective transporters or to dopaminergic and noradrenergic receptors. The drug is rapidly absorbed, with a half-life ranging from 2 to 3 h, while its oral bioavailability remains low. It is primarily metabolized by the hepatic enzyme carboxylesterase 1 (CES1) into an inactive metabolite, ritalinic acid. Consequently, medications that affect this enzyme, as well as ethanol, may alter the pharmacokinetics of methylphenidate and potentially inhibit its action [2].

Methylphenidate exerts a significant effect on cognitive enhancement [1] and is clinically effective in reducing symptoms in patients diagnosed with narcolepsy and ADHD [4], where it remains the first-line pharmacological treatment for both children and adults [1]. This therapy has been shown to lower the risk of suicide, depression, and motor vehicle accidents among adults, as well as all-cause mortality. Additionally, evidence suggests that early stimulant treatment for ADHD may reduce the likelihood of future substance abuse, alcohol dependence, or smoking. However, due to its biochemical similarity to methamphetamine, methylphenidate is often used recreationally, without medical indications, to enhance concentration during studying [5].

Adverse effects pose a significant concern for many patients, with common side effects of psychostimulant medications being dose-dependent and including abdominal pain, appetite suppression, headaches, insomnia, exacerbation of tic disorders, substantial blood pressure fluctuations, tachycardia, and dyskinetic movements. In rare cases, psychotic symptoms may also occur [2,5]. Furthermore, methylphenidate can affect sexual function, leading to dysfunctions in both sexes that may induce stress in patients or strain their relationships with partners [6].

Sexual dysfunction significantly reduces the quality of life for many patients, often causing sufficient distress to warrant a clinical diagnosis of sexual disorder. The primary neurochemical systems involved in the modulation of sexual function include dopamine, norepinephrine, melanocortin, and oxytocin pathways, acting within hypothalamic and limbic brain regions responsible for sexual arousal, attention, and sexual behaviour. The dopaminergic system plays a fundamental role in sexual arousal through the mesolimbic, nigrostriatal, and hypothalamic pathways [7]. Sexual dysfunctions may occur at any stage of the sexual response cycle and are influenced by numerous factors, including medical and psychiatric conditions, cultural background, and life stage [8].

Most sexual dysfunction diagnoses require symptoms to persist for at least six months and occur in approximately 75% of sexual encounters to be classified as a disorder. Eight specific diagnostic categories have been identified: delayed ejaculation, erectile dysfunction, female orgasmic disorder, female sexual interest/arousal disorder, genito-pelvic pain/penetration disorder, male hypoactive sexual desire disorder, premature (early) ejaculation, and substance/medication-induced sexual dysfunction [9]. Erectile dysfunction is the most extensively studied sexual disorder, with an estimated global prevalence ranging from 10% to 20%. Racial, ethnic, and geographical analyses confirm that one in five men experiences this condition [10].

Studies indicate that sexual dysfunction is common among psychiatric patients, likely due to the combined effects of psychopathology and prescribed pharmacotherapy on sexual function. Medications used in psychiatric treatment may indeed impair sexual function and contribute to reduced adherence to pharmacological therapy. Furthermore, sexual dysfunction itself can negatively impact mental health [7].

Dissatisfaction with sexual life and the presence of treatment-related side effects are among the most frequently cited reasons for discontinuing pharmacotherapy [6]. Sexual dysfunction in patients can drastically impair quality of life and, when combined with treatment cessation, may exacerbate underlying psychiatric disorders [8]. Therefore, it is crucial for clinicians to tailor drug dosing to minimize the risk of sexual dysfunction. Patient education during therapy is also essential to prevent treatment discontinuation. Long-term studies on the safety profile of methylphenidate remain an unmet medical need. Consequently, the objective of this study was to conduct a systematic review of the available literature to assess the impact of methylphenidate on patients’ sexual health, identify specific types of sexual dysfunction, and analyse the correlations between psychiatric disorders, treatment dosages and durations, and the presence of sexual dysfunction. Additionally, we aimed to evaluate the prevalence of improved sexual function resulting from methylphenidate use.

## 2. Materials and Methods

This article was prepared in accordance with the PRISMA (Preferred Reporting Items for Systematic Reviews and Meta-Analyses) guidelines [11]. The study was registered in the PROSPERO database under the registration number CRD420250654870.

### 2.1. Inclusion and Exclusion Criteria

The review included studies describing patients of all ages and both sexes. To be eligible for analysis, articles had to provide a detailed description of methylphenidate pharmacotherapy, specifying the administered dose and release formulation. No exclusion criteria were applied regarding comorbid psychiatric disorders to ensure greater representativeness of the study population. Articles describing ADHD patients were not required to include details of the diagnostic process, as ADHD itself was not the primary focus of this analysis.

Only studies in which patients were randomly assigned to groups receiving pharmacological doses of methylphenidate were considered, excluding cases of misuse or overdose. Furthermore, studies had to be published in English and be available in full-text form. The analysis included randomized controlled trials (RCTs) comparing methylphenidate with placebo, as well as cohort studies and case reports.

### 2.2. Search Strategy

A comprehensive literature search was conducted independently by two reviewers. The PubMed database was searched using the following keywords: Sexual dysfunction; sexual disorder; desire; sexual arousal; orgasmic disorder; anorgasmia; sexual pain; erectile disorder; ejaculation; sexual health; delayed ejaculation; premature (early) ejaculation; vulvodynia; dyspareunia; sexual activity; vaginismus; sexual drive; libido; sexual functions; sex life AND “methylphenidate”. In addition to PubMed, we extended our search to include Scopus, Embase, Cochrane Library, and Web of Science. We also searched Google Scholar and grey-literature sources to ensure a comprehensive overview of relevant studies. The first 200 Google Scholar results were screened by title and abstract. Furthermore, ClinicalTrials.gov and other clinical trial registries were searched using the same strategy. No additional eligible articles or ongoing studies meeting our inclusion criteria were identified.

### 2.3. Eligible Studies

The PubMed database search yielded an initial pool of 186 potentially eligible studies. A review of the reference lists of the initially retrieved articles did not identify any additional studies. After screening titles and abstracts, 70 duplicate studies were excluded, and 79 articles were removed based on the inclusion criteria. Of the remaining 37 studies, 23 were further excluded due to a lack of relevant results or content unrelated to the research topic upon full-text review.

**Figure 1:**
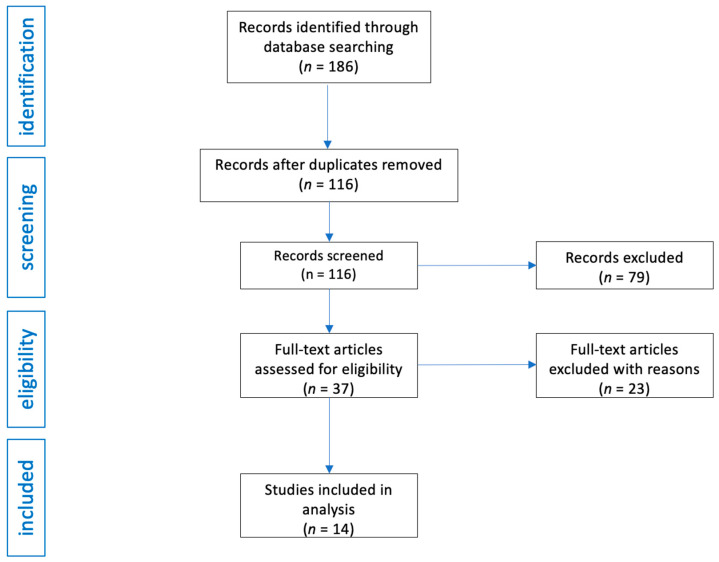
Flow diagram of study analysis and selection for review.

The study selection process is illustrated in Figure 1.

### 2.4. Data Extraction

Key information extracted from the included studies encompassed the first author’s name, the study type, the therapeutic agent used, the ages of participants, the sample size, the characteristics of the intervention, and the quality assessment of the study according to the QATQS Global Rating tool. Particular attention was given to the qualitative analysis of each study during data collection.

### 2.5. Data Quality

The Quality Assessment Tool for Quantitative Studies (QATQS), developed under the Effective Public Health Practice Project (EPHPP) [12,13], was employed to assess the methodological quality of quantitative studies and the potential risk of bias. This tool allows for a comprehensive evaluation of studies conducted under various research designs, including randomized controlled trials (RCTs), observational studies with or without control groups, and case studies, facilitating comparisons of their methodological rigor.

The QATQS consists of eight distinct sections assessing factors such as selection bias, study design, confounding variables, blinding, data collection methods, participant withdrawals and dropouts, intervention integrity, and data analysis. Each section is rated on a three-point scale, where a score of 1 indicates strong quality, 2 moderate quality, and 3 weak quality. The overall study rating is determined by the number of sections rated as weak.

A study is classified as strong if no sections receive a weak rating, moderate if one section is rated weak, and weak if two or more sections receive weak ratings. This systematic approach enables the identification of methodological limitations and ensures an objective evaluation of study quality, which is crucial for the accurate interpretation of findings. Two reviewers independently evaluated each study using the QATQS tool. As both reviewers reached the same ratings in all cases, no discrepancies required resolution and formal inter-rater reliability statistics were not calculated. All key information and synthesized data from the included articles are presented in Table 1.

## 3. Results

### 3.1. Impact on Libido

Methylphenidate, a dopamine and norepinephrine reuptake inhibitor, exerts a complex influence on libido, which has been investigated in various pharmacological contexts. Contrary to the common assumption that psychostimulants significantly enhance sexual drive, research findings indicate a more nuanced effect, dependent on the route of administration, dosage, and experimental conditions. In one study, intravenous administration of methylphenidate was observed to enhance subjective sexual desire, whereas oral administration of a moderate 20 mg dose did not yield similar effects [21]. In a randomized controlled trial involving 30 adults, a 40 mg dose resulted in increased sexual arousal in response to explicit visual stimuli, suggesting that plasma drug concentration may play a pivotal role in modulating libido. This mechanism is likely associated with the drug’s impact on the dopaminergic system, which is integral to motivation and reward processing, including sexual behaviour. However, it is important to acknowledge that these effects were documented under controlled laboratory conditions, where participants were exposed to visual stimuli. Such conditions may not fully reflect real-life circumstances [18].

In a longitudinal study by Edvinsson and Ekselius (2018) [27], which included adults diagnosed with ADHD who were followed for an average of six years, 17% of the 112 patients who continued methylphenidate treatment reported decreased libido as an adverse effect. This finding suggests that, although the drug may attenuate sexual drive in a subset of patients, this side effect was not severe enough to prompt treatment discontinuation in most cases, indicating relative tolerability.

A case report involving a 24-year-old male student illustrates the intricate interplay between ADHD pharmacotherapy, sexual function, and hormonal balance, particularly testosterone levels [26]. The patient, who had exhibited difficulties with concentration, hyperactivity, and anxiety since childhood, experienced significant cognitive improvements following the initiation of methylphenidate at a daily dose of 40 mg. However, this treatment was associated with an undesired decline in libido, negatively impacting the patient’s quality of life and interpersonal relationships. Discontinuation of methylphenidate led to an immediate restoration of sexual drive but resulted in a deterioration of ADHD symptoms. Laboratory assessments revealed normal total testosterone levels but reduced free testosterone, potentially explaining the observed decrease in libido. Consequently, transdermal testosterone therapy was introduced, leading to substantial improvement in both sexual function and ADHD symptoms. Interestingly, testosterone monotherapy not only alleviated impulsivity and inattention but also enhanced mood, motivation, and sleep quality. The patient additionally reported a reduction in seasonal affective disorder symptoms, which had previously been problematic.

### 3.2. Impact on Erectile Function

Methylphenidate has been associated with unexpected alterations in sexual activity, including spontaneous erections. A case report described a patient receiving 18 mg/day of OROS MPH who experienced up to ten spontaneous, non-stimulus-induced erections per day, lasting between five and ten minutes, primarily occurring a few hours post-administration. Although the patient did not report increased sexual arousal or hypersexuality, these episodes caused considerable distress, particularly due to concerns about their visibility to others. When the dose was increased to 36 mg/day, the nature of the erections changed, becoming more closely associated with sexual stimuli and fantasies. Given the distressing nature of these effects and the suboptimal improvement in concentration, treatment was ultimately discontinued, leading to the resolution of symptoms within a few days [23].

Another report documented a 13-year-old patient who experienced multiple episodes of priapism while receiving 54 mg/day of methylphenidate, one of which lasted 17 h and necessitated medical intervention. Doppler ultrasound examination revealed low-flow priapism, attributed to chronic occlusive changes and vasospasms within the corpora cavernosa. The condition was managed through aspiration and irrigation, leading to symptom resolution; however, treatment discontinuation was required [16].

A similar case involved an 8-year-old patient with ADHD receiving extended-release methylphenidate (54 mg/day) in combination with risperidone (1 mg/day), who developed painful priapism in the absence of sexual stimulation. The first episode occurred on day 22 of combined therapy and required emergent intervention, including cavernosal aspiration and intracavernosal epinephrine infusion. Despite initial symptom relief, two additional episodes occurred during hospitalization, though further surgical interventions were not necessary. Following the discontinuation of both medications, priapism resolved completely within seven days. The symptoms were attributed to a potential interaction between the two drugs, as no predisposing vascular conditions were identified. Subsequently, ADHD treatment was switched to atomoxetine (50 mg/day), which resulted in symptom improvement without recurrence of priapism, corroborating the hypothesis that psychostimulants and antipsychotics played a causative role in its onset [17].

An additional case described a preschool-aged patient who similarly developed priapism while undergoing methylphenidate treatment for ADHD. The condition necessitated medical intervention, including cavernosal drainage and topical pharmacological management. Ultimately, the priapism was linked to methylphenidate therapy, leading to treatment discontinuation. This case underscores the importance of early recognition of rare but potentially serious adverse effects associated with methylphenidate, highlighting the necessity for individualized pharmacological management in paediatric patients, particularly those at risk of vascular complications [22].

### 3.3. Impact on Ejaculation and Semen Parameters

Methylphenidate has been implicated in disturbances of ejaculatory function, as evidenced by a case involving a 16-year-old male with ADHD [24]. The patient, who was prescribed 30 mg/day of MPH, experienced spontaneous ejaculations that were unrelated to erection or sexual arousal and were concomitant with episodes of heightened anxiety. Following drug discontinuation, these symptoms resolved, suggesting a causal relationship between methylphenidate use and this uncommon side effect. The underlying mechanism may be associated with the drug’s role as a norepinephrine reuptake inhibitor, given norepinephrine’s crucial involvement in both central and peripheral ejaculatory pathways [28].

Methylphenidate may also influence semen parameters, particularly when administered long-term from adolescence. Its mechanism of action, which involves increasing dopamine and norepinephrine neurotransmission, has been linked to potential effects on spermatogenesis and ejaculatory function [29]. Animal studies have demonstrated that methylphenidate and its analogues can induce apoptotic activity in seminiferous tubules, reduce testicular mass, and impair sperm motility and count [30]. In humans, cases of azoospermia and idiopathic testicular insufficiency have been reported in individuals with prolonged MPH use from early childhood [31].

Clinical studies suggest that stimulant medications may decrease semen volume and total motile sperm count, although this effect does not necessarily stem from direct spermatotoxicity but rather from adrenergic mechanisms such as desensitization of sympathetic adrenergic receptors [32]. Chronic elevation of catecholamine levels due to prolonged sympathomimetic use may impair seminal fluid emission and bladder neck closure, negatively affecting ejaculatory function. While these effects may be clinically insignificant in individuals with normal semen parameters, they could have implications for patients with already reduced total motile sperm counts, particularly in the context of assisted reproductive techniques such as intrauterine insemination or in vitro fertilization [25].

### 3.4. Impact on Sexual Behaviours

Studies have demonstrated that individuals with ADHD report heightened sexual desire, increased frequency of masturbation, and lower sexual satisfaction, compared with the general population. Current evidence suggests a link between elevated impulsivity in individuals with ADHD and hypersexuality [33]. In a documented case of a paediatric patient presenting with both ADHD symptoms and early puberty, treatment with methylphenidate (MPH) led to significant improvements in ADHD-related behaviours, including hyperactivity, impulsivity, and attentional difficulties. One month after initiating therapy, a marked reduction in behavioural symptoms was observed, as reflected in a decreased ADHD-IV rating scale score [19]. Despite this improvement, the patient’s sexual drive was not significantly attenuated by MPH, indicating the drug’s limited efficacy in modulating heightened sexual functions.

Case analysis suggests that the patient’s sexual drive was closely associated with transient hyperandrogenism. Elevated androgen levels, documented in this patient, may have contributed to increased sexual desire, which only subsided following the introduction of a gonadotropin-releasing hormone (GnRH) analogue—a highly effective therapy for suppressing hypothalamic–pituitary–gonadal axis activity. This phenomenon underscores the distinct mechanism of action of MPH, which, in contrast to GnRH analogues, does not directly modulate hormonal balance or androgen levels. Furthermore, the use of MPH should be approached with caution in paediatric patients.

A case report in the literature describes the significant impact of methylphenidate on sexual and masturbatory behaviours in a 7-year-old girl diagnosed with ADHD [20]. Following the initiation of MPH at a dose of 5 mg twice daily, the patient began engaging in manual genital stimulation. Masturbatory episodes occurred two to three times per day, lasting approximately ten minutes each. Initially, this behaviour was not attributed to the medication; however, upon increasing the MPH dose to 10 mg/day, ADHD symptoms improved, while masturbatory behaviours intensified further. The child engaged in masturbation for two to three hours daily, leading to vulvar bleeding. Additionally, she exhibited increased interest in sexual topics and displayed her legs and underwear to male peers. These behaviours significantly diminished during weekends and other periods when MPH was not administered. Upon discontinuation of MPH, ADHD symptoms worsened, yet sexual and masturbatory behaviours rapidly decreased, ultimately resolving within one week. During the follow-up period after cessation of MPH therapy, these behaviours did not re-emerge. This case highlights the potential of psychostimulants to induce hypersexuality and excessive masturbation in prepubertal children.

### 3.5. Impact on Impaired Sexual Function in Patients with Depression

In patients experiencing sexual dysfunction coexisting with depression, determining the primary cause of dysfunction is critical, as both depressive illness and its pharmacological treatment can contribute to such disturbances. In one reported case, depressive symptoms that had progressively worsened over three years were accompanied by loss of libido and erectile dysfunction, with a notable exacerbation of these issues over the preceding six months. Based on this progression, depression was identified as the primary aetiology of the patient’s sexual dysfunction. Initial treatment with selective serotonin reuptake inhibitors (SSRIs) and serotonin-norepinephrine reuptake inhibitors (SNRIs) was ineffective, despite dose optimization. Significant symptomatic relief was achieved only after the introduction of vortioxetine at 10 mg/day, as evidenced by a reduction in the Hamilton Depression Rating Scale score to 5 points within two months. However, libido loss and erectile dysfunction persisted, adversely affecting the patient’s quality of life. The addition of short-acting methylphenidate at 10 mg/day led to a marked improvement in sexual function. Within one week of treatment, the patient reported increased libido and resolution of erectile difficulties during sexual activity. Furthermore, MPH therapy mitigated excessive daytime sleepiness and enhanced attention and concentration, suggesting its broader role in supporting both psychological and physiological functioning [14].

A separate case involved a 28-year-old male diagnosed with a severe depressive episode at the age of 23, whose pharmacological treatment significantly impacted his sexual function. Despite previously reporting a highly satisfying sex life and no history of sexual dysfunction, sequential antidepressant treatments—including paroxetine, sertraline, venlafaxine, and milnacipran—progressively impaired his sexual function. These adverse effects manifested as weak erections, reduced genital sensitivity, anorgasmia, and complete loss of libido, reaching their peak during milnacipran therapy. Even after discontinuation of all antidepressants, residual effects such as anhedonic ejaculation, absence of sexual desire, and a lack of response to sexual stimuli persisted, despite the absence of identifiable physical, biochemical, or psychological causes.

Extended-release methylphenidate (54 mg once daily) resulted in a significant improvement in sexual function. MPH therapy led to increased libido, restored the patient’s response to sexual stimuli, and enhanced his ability to experience pleasure during sexual activity. These effects were more pronounced than those observed with prior treatments involving dopamine agonists (pramipexole, ropinirole, cabergoline) and bupropion, which had provided only partial and short-lived relief [15].

The pharmacological mechanism of MPH involves the inhibition of dopamine reuptake in the central nervous system, thereby enhancing dopaminergic neurotransmission. Given the critical role of dopamine in modulating sexual desire and pleasure, this mechanism likely accounts for the observed therapeutic benefits. However, the improvement in sexual function proved transient, as symptoms re-emerged following MPH discontinuation. This underscores the need for further research into long-term strategies for managing sexual dysfunction in this patient population.

## 4. Discussion

The impact of methylphenidate (MPH) on sexual function is complex, and results from its influence on neurotransmitter systems, particularly the dopaminergic and noradrenergic pathways. The ability of MPH to increase dopamine availability in synapses may enhance stimulation of the reward system, which also encompasses sexual arousal [34]. However, unlike stimulant substances such as cocaine or methamphetamine, which are frequently associated with risky sexual behaviours, methylphenidate appears to induce more moderate effects [21]. It has been observed that this drug may interfere with the mechanisms of conditioned inhibition of sexual arousal, which could explain its influence on libido. Furthermore, studies in which patients were exposed to both implicit and explicit sexual stimuli found that MPH significantly increased sexual arousal only in response to explicit visual content, suggesting that its effects in this domain may depend on the nature of the stimulus and the context in which it is perceived [18].

Similarly to other agents with noradrenergic activity, MPH may affect the sympathetic nervous system, potentially reducing ejaculation latency [28]. This effect aligns with observations concerning norepinephrine reuptake inhibitors, such as milnacipran and reboxetine, which also modulate physiological processes related to ejaculation [35]. There are reports of spontaneous ejaculation in patients receiving MPH, particularly during periods of heightened stress, which may result from interactions between sympathetic nervous system activity and increased anxiety. In such cases, a potential therapeutic strategy could involve the use of selective serotonin reuptake inhibitors (SSRIs) which, due to their ability to inhibit the orgasmic phase, may counteract these adverse effects [36]. Paradoxically, MPH has also demonstrated the ability to reverse SSRI-induced sexual dysfunctions [37], further complicating understanding of its overall impact on sexual function and suggesting the involvement of multiple mechanisms. The bidirectional effects on libido may stem from dose-dependent neuroadaptations. Acute high-dose MPH (e.g., intravenous) transiently enhances dopaminergic signalling, whereas chronic use downregulates receptors, reducing sexual drive. Comorbidities like diabetes (vascular dysfunction) or neuropathy (sensory deficits) likely modulate these effects. Future studies should stratify outcomes by dose, duration, and metabolic profiles. Additionally, hormonal factors may modulate individual responses to MPH treatment, as evidenced by research on testosterone levels and paediatric patients.

Although not directly assessed in the included studies, patients with MPH-induced sexual dysfunction may seek over-the-counter supplements such as L-arginine, ginseng, or maca root to alleviate symptoms. These agents are believed to enhance nitric oxide-mediated vasodilation or modulate endocrine responses. For instance, L-arginine supplementation has been associated with increased nitric oxide levels, leading to improved vasodilation and potential benefits in erectile function [38]. Ginsenosides, the active components of Panax ginseng, have been shown in animal studies to promote nitric oxide release, facilitating relaxation of the corpus cavernosum and enhancing copulatory behaviour [39]. Similarly, maca root (*Lepidium meyenii*) has been traditionally used to improve sexual function, although evidence from systematic reviews remains limited [40]. However, the efficacy and safety of these supplements in individuals receiving MPH remain uncertain, and interactions with dopaminergic or adrenergic systems cannot be excluded. Future studies should consider evaluating the impact of such supplements in this patient population.

The sexual effects of methylphenidate should be considered in the context of other stimulants, such as cocaine and amphetamines. Cocaine, a potent dopamine transporter blocker, is frequently associated with heightened libido, risk-taking sexual behaviour, and increased frequency of intercourse, especially in recreational settings. However, its use is also associated with erectile dysfunction and delayed ejaculation, likely due to vasoconstriction and prolonged sympathetic activation [41]. In contrast, amphetamines such as methamphetamine initially enhance libido and arousal but may lead to long-term sexual dysfunction due to neurotoxicity and downregulation of dopaminergic pathways [42]. Methylphenidate, while structurally related to amphetamines, tends to produce more moderate and context-dependent effects on sexual function, with fewer reports of impulsive or risky sexual behaviours. This difference may reflect its pharmacokinetic profile, lower dopaminergic potency, and regulated medical use.

The long-term impact of MPH on fertility and semen parameters remains an insufficiently explored area. Current data suggest that decisions regarding treatment modifications aimed at optimizing reproductive function should be individualized, balancing the patient’s mental health needs with reproductive concerns. In cases where temporary discontinuation of the drug is considered, its half-life—ranging from 4 to 7 h for MPH and from 10 to 15 h for amphetamine derivatives—should be considered [43]. While some observed changes may be reversible, the current state of knowledge does not allow for definitive conclusions regarding the long-term consequences of stimulant use on reproductive health, underscoring the necessity for further research.

Particularly concerning are reports of sexual manifestations in children receiving chronic MPH therapy for ADHD. Case reports in the literature describe excessive masturbation in preschool and early school-aged children, as well as priapism in boys, highlighting the potential disruption of sexual development—a complex and multifaceted process influenced by biological, psychological, and social factors [44].

This systematic review has several significant limitations. First, most of the included studies are case reports, which limit the generalizability of findings. It is important to note that the majority of included case reports received a QATQS rating of 3 (weak), which considerably limits the level of evidence. As a result, conclusions drawn from these reports should be interpreted with caution and be considered primarily as exploratory or hypothesis-generating. Additionally, the considerable variation in MPH dosages and formulations, ranging from 5 mg to 100 mg, complicates the interpretation of results and the identification of precise mechanisms underlying its effects on sexual function. The study population encompasses children, adolescents, and adult males, further challenging result interpretation, particularly regarding the influence of puberty and hormonal maturation. Moreover, long-term studies assessing the persistence of sexual symptoms after prolonged MPH therapy are lacking. Finally, the review primarily focuses on the pharmacological effects of MPH without fully considering psychological and environmental factors that may contribute to patients’ sexual development. A critical gap in current research is the absence of paediatric randomized controlled trials investigating long-term reproductive and sexual development outcomes following methylphenidate therapy. This is particularly important given the increasing prevalence of early pharmacological intervention in children and adolescents with ADHD. Despite expanding our search strategy to include additional databases, grey literature, Google Scholar, and clinical trial registries, a significant overlap in results was observed across sources. While this supports the consistency of the available evidence, it also suggests that potentially relevant studies may have been missed due to limitations in keyword combinations or database indexing. Although we also screened abstracts not indexed in the primary databases, it remains possible that some eligible publications were not captured. This highlights a limitation of our review, and future research may benefit from testing broader or alternative search terms to identify further literature in this emerging field. These limitations emphasize the need for larger, controlled clinical studies to enhance our understanding of the relationship between MPH use and sexual function. The limited and often contradictory nature of the available studies highlights the urgent need for safer and more targeted pharmacological options in ADHD management, especially those with a reduced risk of sexual side effects.

## 5. Conclusions

In summary, the effects of methylphenidate on sexual function are multifaceted and influenced by various factors, including neurochemical, hormonal, and contextual mechanisms. While this medication may contribute to increased sexual arousal under specific conditions, it can simultaneously lead to ejaculatory disturbances. Therefore, monitoring sexual function should be an integral part of long-term follow-up in patients receiving MPH therapy, as impairments in this domain may diminish quality of life and increase the risk of non-adherence to treatment. Future research should focus on elucidating the mechanisms underlying these effects and developing strategies to mitigate them, which could be crucial for improving the well-being of individuals undergoing psychostimulant therapy. Further high-quality research is needed to determine the long-term safety of methylphenidate on sexual health and to develop improved treatment alternatives that maintain therapeutic efficacy while minimizing adverse effects.

## Figures and Tables

**Table 1:** Summary of included studies assessing the impact of methylphenidate on sexual function. This table presents the type of article, patient characteristics, the type of pharmacotherapy, observed effects on sexual function, and the methodological quality of each study assessed using the Quality Assessment Tool for Quantitative Studies (QATQS) from the Effective Public Health Practice Project (EPHPP). The overall quality rating for each publication was assigned as follows: 1—strong, 2—moderate, 3—weak.

Author	Type of Article	Patients	Pharmacotherapy	Impact on Sexual Life	QATQS Assessment
Şerif Bora Nazlı [14]	Case report	40-year-old man	10 mg immediate-release MPH	Increased libido	3
Antonei B Csoka [15]	Case report	28-year-old man	54 mg extended-release MPH	Erectile dysfunction	3
Muharrem Burak Baytunca [16]	Case report	13-year-old boy	10–54 mg immediate-release MPH	Priapism	3
Hatice Unver [17]	Case report	12-year-old boy	54 mg extended-release MPH	Priapism	3
Jasmin Schmid [18]	Controlled clinical trial	30 adults	40 mg immediate-release MPH	Increased libido	3
Koujyu Katayama [19]	Case report	9-year-old boy	18–27 mg of extended-release MPH	No inhibition of sexual desire	3
Ayhan Bilgiç [20]	Case report	7-year-old-girl	5 mg MPH twice a day	Frequent masturbation	3
Nora D. Volkow [21]	Randomized trial	39 men	0.5 mg/kg intravenous MPH	Increased libido	3
Ozalp Ekinci [22]	Case report	5-year-old boy	10 mg immediate-release MPH	Priapism	3
Murat Coskun [23]	Case report	15-year-old-boy	36 mg immediate-release MPH	Spontaneous erection 10 times a day	3
Bedriye Öncü [24]	Case report	16-year-old boy	30 mg immediate -release MPH	Spontaneous ejaculations	3
Minh N Pham [25]	Cohort study	8861 men	various forms of MPH	Deterioration in fertility and semen quality	3
Ane Rogne [26]	Case report	24-old man	40 mg extended-release MPH	Decreased libido	3
Dan Edvinsson [27]	Cohort study	168 participants	10–100 mg immediate-release MPH	Decreased libido	2
